# Evaluation of the OsTIR1 and AtAFB2 AID Systems for Genome Architectural Protein Degradation in Mammalian Cells

**DOI:** 10.3389/fmolb.2021.757394

**Published:** 2021-11-04

**Authors:** Anastasia Yunusova, Alexander Smirnov, Tatiana Shnaider, Varvara Lukyanchikova, Svetlana Afonnikova, Nariman Battulin

**Affiliations:** ^1^ Institute of Cytology and Genetics SB RAS, Novosibirsk, Russia; ^2^ Department of Natural Sciences, Novosibirsk State University, Novosibirsk, Russia

**Keywords:** auxin-inducible degron (AID) system, CRISPR/Cas9, condensin, cohesin, mouse ES cells, HAP1 cells

## Abstract

The auxin-inducible degron (AID) system is a promising tool for dynamic protein degradation. In mammalian cells, this approach has become indispensable to study fundamental molecular functions, such as replication, chromatin dynamics, or transcription, which are otherwise difficult to dissect. We present evaluation of the two prominent AID systems based on OsTIR1 and AtAFB2 auxin receptor F-box proteins (AFBs). We analyzed degradation dynamics of cohesin/condensin complex subunits in mouse embryonic stem cells (Rad21, Smc2, Ncaph, and Ncaph2) and human haploid HAP1 line (RAD21, SMC2). Double antibiotic selection helped achieve high homozygous AID tagging of an endogenous gene for all genes using CRISPR/Cas9. We found that the main challenge for successful protein degradation is obtaining cell clones with high and stable AFB expression levels due to the mosaic expression of AFBs. AFB expression from a transgene tends to decline with passages in the absence of constant antibiotic selection, preventing epigenetic silencing of a transgene, even at the *AAVS1* safe-harbor locus. Comparing two AFBs, we found that the OsTIR1 system showed weak dynamics of protein degradation. At the same time, the AtAFB2 approach was very efficient even in random integration of AFB-expressed transgenes. Other factors such as degradation dynamics and low basal depletion were also in favor of the AtAFB2 system.

## Introduction

Loss-of-function experiments reveal important information about molecular pathways and gene functions. For most critical genes, whose knockout is lethal for cells, temporal knockdown methods, such as RNA interference, are employed, but they could be ineffective. The auxin-inducible degron (AID) system has provided research community with a unique tool for rapid and substantial degradation of a protein of interest (POI), controlled by the addition of an auxin molecule (Nishimura et al., 2009). The AID system has been successfully applied in many organisms ranging from yeast to transgenic mice (Yesbolatova et al., 2020). Adaptation of the AID system in popular cell cultures, such as HeLa, HCT116, mouse and human stem cells, and neurons (Natsume et al., 2016; Nora et al., 2017; Nakano et al., 2019; Aksenova et al., 2020; Pryzhkova et al., 2020; Thiecke et al., 2020), was extremely fruitful, which allowed to study complex molecular pathways for virtually unlimited applications.

To perform AID experiments, cells should be modified with specific genetic constructs. First, a short peptide AID domain is fused via endogenous modification to a protein of interest (POI) at the N- or C-terminus. In addition, an auxin receptor F-box protein (AFB) is expressed to form a ubiquitin E3 ligase complex with other cellular components. The AFB recognizes the AID domain of the chimeric POI in the presence of the auxin molecule [indole-3-acetic acid (IAA) or other analogs], which leads to polyubiquitination and fast proteasomal degradation of the POI, usually over the course of just a few hours.

Researchers have employed several AID systems using different AFBs, of which *Oryza sativa* TIR1 (OsTIR1) (Yesbolatova et al., 2019) has become the most popular choice and *Arabidopsis thaliana* AFB2 (AtAFB2) has come to prominence in recent years (Li et al., 2019). OsTIR1 facilitates degradation of POI tagged with the miniAID domain (amino acids 68–132 of *Arabidopsis thaliana* IAA17 protein), while AtAFB2 interacts with the miniIAA7 domain (amino acids 37–104 of the same protein), leading to similar results. Experimental data show that the AtAFB2 approach could be preferential due to faster degradation dynamics and lack of basal degradation (depletion of the POI in the absence of auxin) (Li et al., 2019).

During last years, researchers have come up with several strategies to generate stable transgenic cell lines expressing the AID system. But even though many great protocols exist (Lambrus et al., 2018; Yesbolatova et al., 2019; Sathyan et al., 2020), the method still contains pitfalls that could obstruct new users. For instance, selection of the proper transgenic clone with high POI degradation efficiency could be unmanageable due to AFB silencing. We decided to share our experience of using two popular auxin degron systems to help readers avoid some common difficulties.

In our experiments, we used the AID system to study the effects of condensin/cohesin complex depletion on the nuclear architecture in two popular cell types: mouse embryonic stem cells (mESCs) and human haploid HAP1 cell line. For this purpose, we tagged mouse *Rad21*, *Smc2*, *Ncaph*, and *Ncaph2* genes and human *RAD21*, *SMC2* genes with miniAID tag (OsTIR1 system) (Natsume et al., 2016) or miniIAA7 tag (AtAFB2 system) (Li et al., 2019), transfected cells with corresponding AFBs, and analyzed protein degradation dynamics, using microscopy, FACS, and western blotting.

## Results

### Gene Locus Targeting With Auxin-Inducible Degron Cassette in mES and HAP1 Cells

**FIGURE 1:**
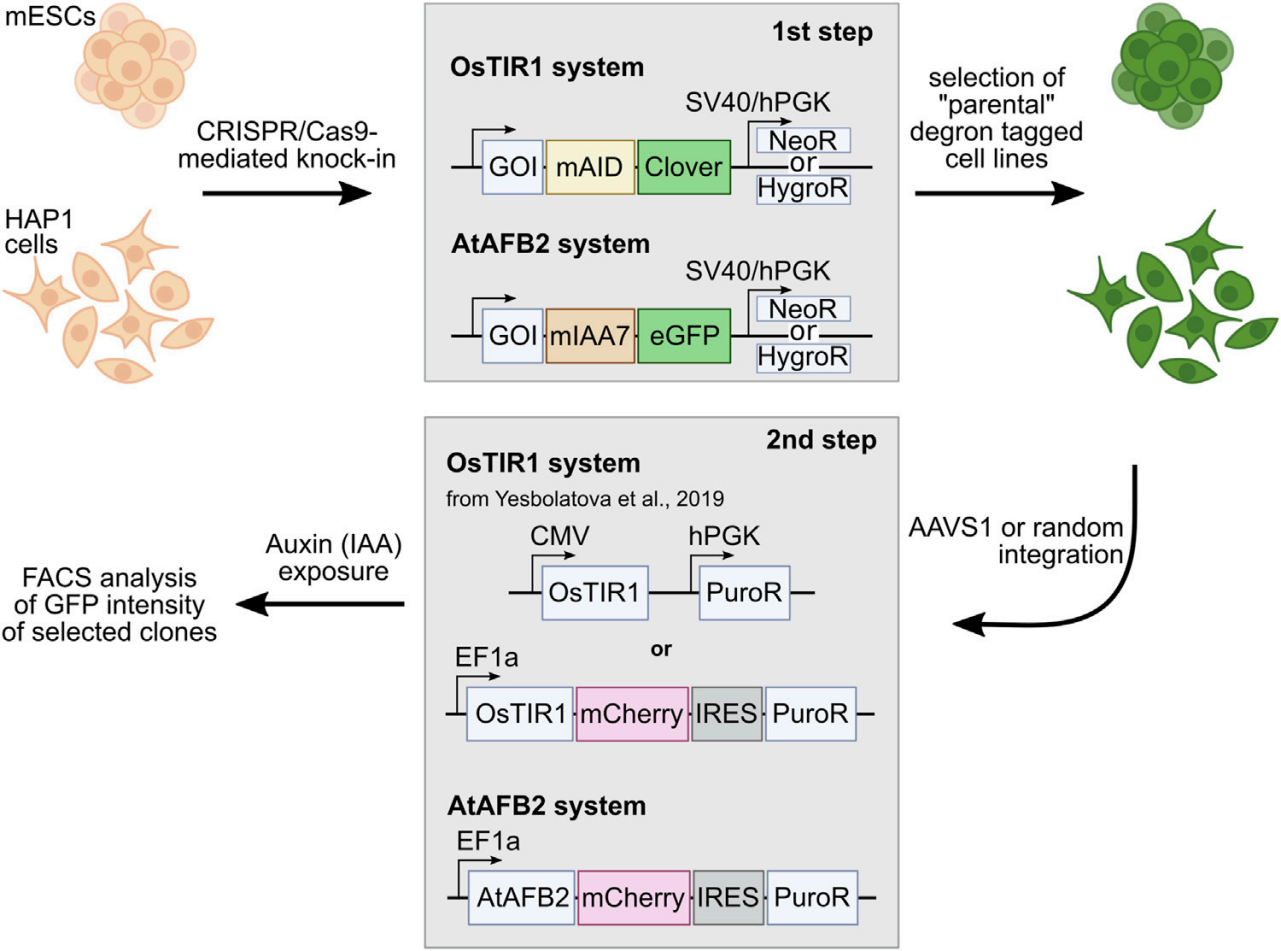
Outline of the AID experiments performed with two AID systems: OsTIR1 and AtAFB2. The first step includes targeted integration of the AID tags and *NeoR*/*HygroR* selection cassette. In the second step, selected degron-tagged clones are transformed with AFB expression plasmids with the Puro^R^ selection cassette. GOI, gene of interest.

We tagged endogenous target loci with AID tag fused with Clover/eGFP by CRISPR/Cas9-mediated HDR (Natsume et al., 2016; Li et al., 2019). In total, we modified four genes (*Rad21*, *Smc2*, *Ncaph*, and *Ncaph2*) in mES cells and two genes (*RAD21*, *SMC2*) in HAP1 cells (Table 1). We used single gRNA targeting a region around the STOP codon to introduce an AID and selection cassette at the 3′-ends of the genes (Figure 1). Homology-directed integration of the AID cassette was facilitated by 600–1,300 bp homology arms (Supplementary Table S2). In our study, we used a minimal required AID (either mAID or miniIAA7) linked to Clover/eGFP (Figure 1). The donor vector was a mix of the two constructs that contained different selection markers (NeomycinR or HygromycinR). Such a strategy allowed us to select diploid cells that tagged all two alleles efficiently because only cells in which one allele is modified with a construct with a *NeomycinR* marker and the other allele with a *HygromycinR* marker will survive in a medium with both antibiotics (double selection).

**TABLE 1:** Gene targeting efficiencies in mESC and HAP1 clones in the example of the AtAFB2 system.

	Total colonies genotyped	Homozygous targeting
ES-*Rad21*	15	5 (33.3%)
ES-*Smc2*	15	6 (40%)
ES-*Ncaph*	24	6 (25%)
ES-*Ncaph2*	19	5 (26%)
HAP1-*RAD21*	37	4 (11%)
HAP1-*SMC2*	13	10 (77%)

In mESCs, *Rad21* gene targeting resulted in 33% homozygous clones, the *Smc2* gene in 40%, the *Ncaph* gene in 25%, and the *Ncaph2* gene in 26%. In HAP1 cells, *RAD21* gene targeting resulted in 11% homozygous clones and the *SMC2* gene in 77% (Table 1). We also performed second round modification of one of the mESC *Rad21*-miniIAA7-eGFP clones to introduce *Smc2*-miniIAA7-eGFP for double protein degradation. In this case, we could not use Neo/Hygro selection; instead, we visually inspected clones and picked only those with eGFP spread across both the nucleus and the cytoplasm (in *Rad21* clones, eGFP is found in the nucleus) (Supplementary Figure S1).

The correct insertion of the AID cassette was confirmed by long-distance PCR (Supplementary Figure S2) and Sanger sequencing. The molecular weight of fusion proteins was verified by immunoblotting (in case of Rad21 and Smc2). All analyzed clones were positive for Clover/eGFP expression and had a correct protein distribution pattern inside cells, i.e., Rad21-miniIAA7-eGFP and Ncaph2-miniIAA7-eGFP were confined to the nucleus, while Smc2-miniIAA7-eGFP and Ncaph-miniIAA7-eGFP resided predominantly in the cytoplasm (Supplementary Figure S1). At least four homozygous clones were expanded and frozen for each gene modification, and a few (2–3) clones were selected for subsequent transfection with AFB expression vectors.

**FIGURE 2:**
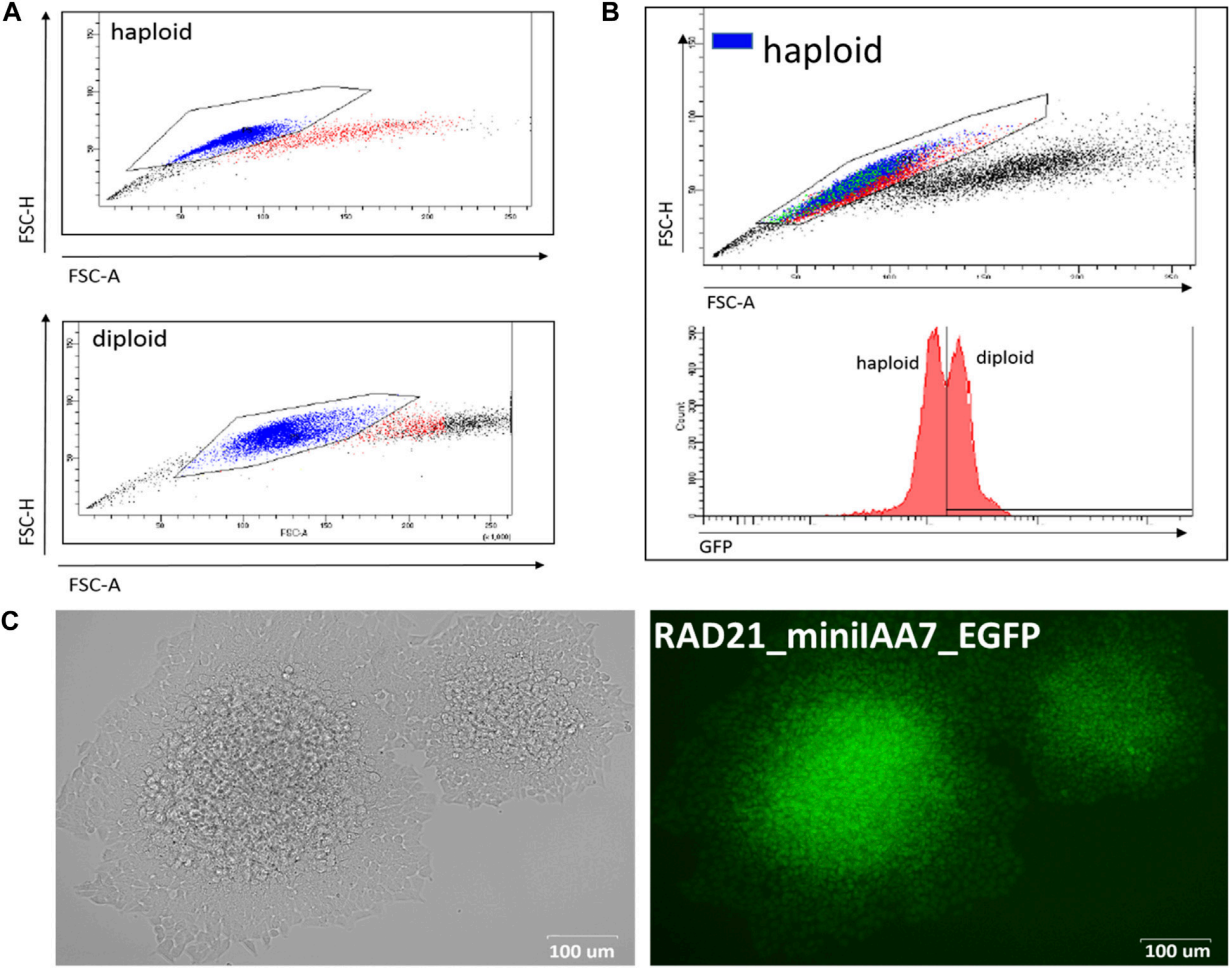
Haploid and diploid HAP1 clones could be reliably separated by cell size using FACS or visual clues. **(A)** FACS plots for haploid and diploid HAP1 clones. **(B)** Mosaic HAP1 clone consisting of two subpopulations in equal proportions. Blue/red highlight indicates haploid/diploid subpopulations, respectively. **(C)** Two eGFP-positive RAD21-miniIAA7-eGFP clones that show significant cell size differences, making it easy to pick the correct clone (haploid clone is on the right).

As far as we know, this is the first application of the AID system in human HAP1 cells. This cell line is a powerful tool for genome engineering because most of the genes are present only in one copy, which simplifies gene modifications. The efficiency of gene targeting was comparable to that of mES cells (Table 1) but required an additional selection step because HAP1 cells have a tendency to spontaneously transit to a diploid state during proliferation (Beigl et al., 2020). Importantly, ploidy shift interferes with targeting experiments and complicates obtaining homozygous clones after AID cassette targeting. Haploid cells could be selected in two ways. First of all, the mixed population of haploid/diploid cells could be reliably sorted by cell size using FACS (Beigl et al., 2020) (Figure 2A; see also Figure 2B, where two subpopulations are highlighted with a color scheme). Alternately, haploid colonies could be found and picked after simple visual inspection of a plate after an antibiotic selection step. Figure 2C demonstrates fluorescent RAD21-miniIAA7-eGFP HAP1 colonies of different ploidy from one of the experiments. HAP1 cell diploidization at the late passages (after AFB transfections) does not seem to affect AID degradation efficiency.

### Evaluation of the Protein of Interest Degradation Efficiency of Two Auxin Receptor F-Box Proteins

**FIGURE 3:**
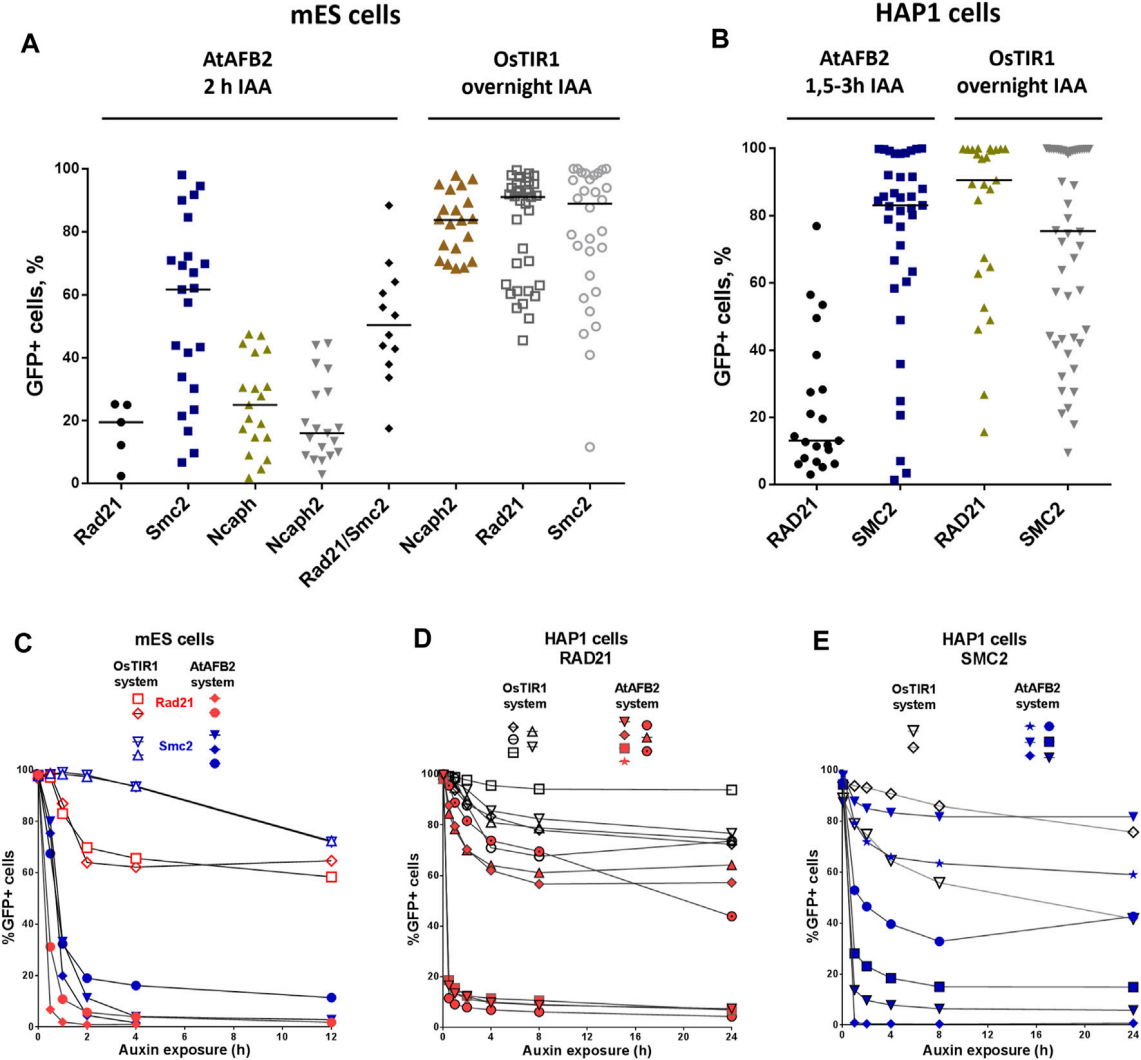
Degradation efficiency and dynamics of POI depletion in mESC and HAP1 clones with randomly integrated OsTIR1 and AtAFB2 constructs. POI degradation efficiency in mESC clones **(A)** and HAP1 clones **(B)**. Each dot represents one independent cell clone derived after AFB protein gene integration. For each clone, the measurement was carried out once. The black horizontal line shows the median of the group. **(C)** POI degradation dynamics for selected mESC clones (Rad21-mAID-Clover, Smc2-mAID-Clover, Rad21-miniIAA7-eGFP, Smc2-miniIAA7-eGFP) with OsTIR1 and AtAFB2. For each clone, the experiment was carried out once. **(D)** POI degradation dynamics for HAP1 clones (RAD21-miniIAA7-eGFP, RAD21-mAID-Clover) with AtAFB2 and OsTIR1. For each clone, the experiment was carried out once. **(E)** POI degradation dynamics for HAP1 clones (SMC2-miniIAA7-eGFP, SMC2-mAID-Clover) with AtAFB2 and OsTIR1. Numbers in brackets are individual clones’ designations. For each clone, the experiment was carried out once.

Selected single homozygous tagged clones (Table 1) were transfected with either CMV-OsTIR1-hPGK-PuroR construct (Natsume et al., 2016) or EF1-AtAFB2-Cherry-IRES-PuroR construct (Li et al., 2019) and cultivated in the presence of puromycin for 1 week. Surviving clones with randomly integrated AFBs were expanded and tested for the POI degradation efficiency on FACS. At this stage, we noted that the two systems show significant degradation of the target protein at different times. Therefore, to characterize clones with the integration of AtAFB2, we performed FACS analysis after 1.5–3 h and for OsTIR1 after overnight incubation in auxin. Plot diagrams show the distribution of Clover/eGFP fluorescence in mESC and HAP1 clones (Figures 3A,B). The OsTIR1 approach using CMV-OsTIR1-hPGK-PuroR was mostly inefficient and required more time to degrade the POI without any actual significant depletion. At the same time, the AtAFB2 transgene, in which the AFB is co-expressed with *PuroR* and *Cherry* genes from a single promoter, allowed to achieve efficient and rapid degradation. Western blot analysis confirmed FACS results for selected clones (Supplementary Figure S3).

Clones with the highest level of protein degradation were selected for further studying the dynamics of this process (data for POI = Rad21 and Smc2). We also included HAP1 clones (AtAFB2 system) with intermediate and low sensitivity to auxin to investigate whether longer auxin exposure can enhance protein depletion efficiency.

Overall, AtAFB2 cell lines displayed more rapid and efficient depletion of the degron-fused proteins (Figures 3C–E). For instance, Rad21 levels significantly diminished after 30 min of auxin exposure (Figures 3C,D), which led to the collapse of nuclear architecture and cell death after 24 h in accordance with earlier results. Although AtAFB2-Cherry protein was mostly localized in the cytoplasm due to weak NLS, it was enough to rapidly degrade nuclear chromatin factors (Li et al., 2019). Moreover, extended time of auxin exposure does not seem to improve protein degradation efficiency: the most pronounced changes in proteins levels occurred during the first 30–120 min and then reached a plateau (Figure 3E).

**FIGURE 4:**
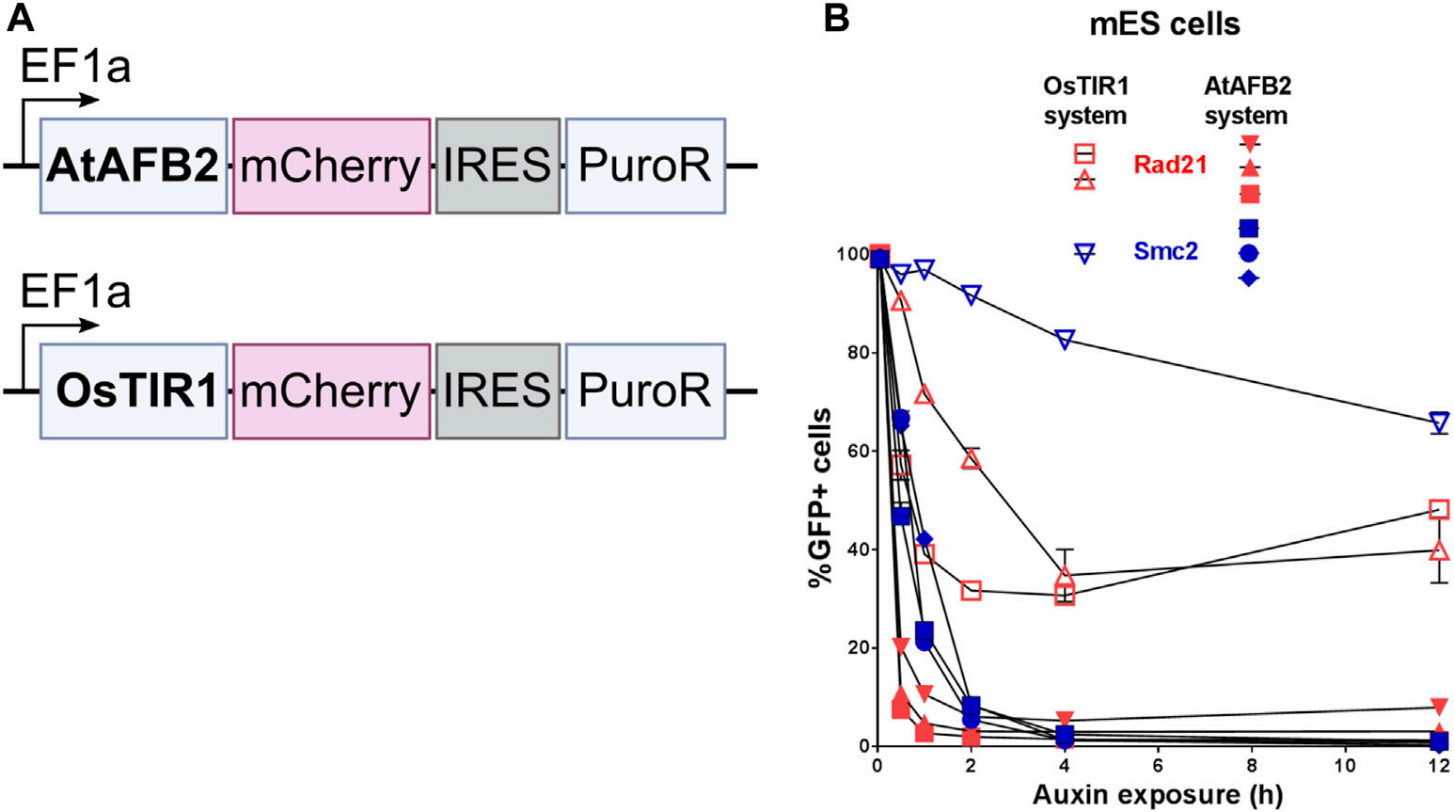
Comparison of degradation efficiency with AFB protein driven by the same EF1a promoter. **(A)** Scheme of genetic constructs used. **(B)** Dynamics of POI depletion in mESC clones (Rad21 colored in red; Smc2 colored in blue). Vectors expressing AFB proteins: pSH-EFIRES-P-OsTIR1-mCherry-weak NLS and pSH-EFIRES-P-AtAFB2-mCherry-weak NLS, were randomly integrated. For this analysis, all clones were grown in puromycin, and the proportion of Cherry-positive cells in each of them was more than 95%. For each clone, the measurement was carried out twice.

OsTIR1 and AtAFB2 systems differ in several components; in particular, they use different AFB proteins, tag sequences, and the expression of the AFB protein driven by different promoters (Figure 1). Because of these differences, it is challenging, if not impossible, to compare these systems in parallel under the same conditions accurately. However, we decided to assess whether the promoter of AFB protein affects the degradation efficiency. To do this, we cloned the coding part of the *OsTIR1* gene instead of AtAFB2 (Figure 4A). Then, we integrated the resulting construct into a random place in the genome of mESC clones with the tagged *Smc2* or *Rad21* gene. For each gene, we picked three random colonies and tested the degradation efficiency. In one clone of *Rad21* and in two clones of *Smc2*, there was no degradation at all. For the rest, we examined the dynamics of degradation. None of the clones tested showed more than 75 percent degradation (Figure 4B). These data may indicate that the difference between the systems from the two articles is not due to the promoter providing greater expression in the AtAFB2 system.

The AtAFB2 construct contains an in-frame Cherry fluorescent marker which is extremely useful to select suitable clones, since Cherry levels reflect AtAFB2 expression. On FACS, we noted a strict correlation between Cherry expression and POI degradation efficiency for the eGFP signal (data not shown).

### Silencing of the Auxin Receptor F-Box Protein Transgene

**FIGURE 5:**
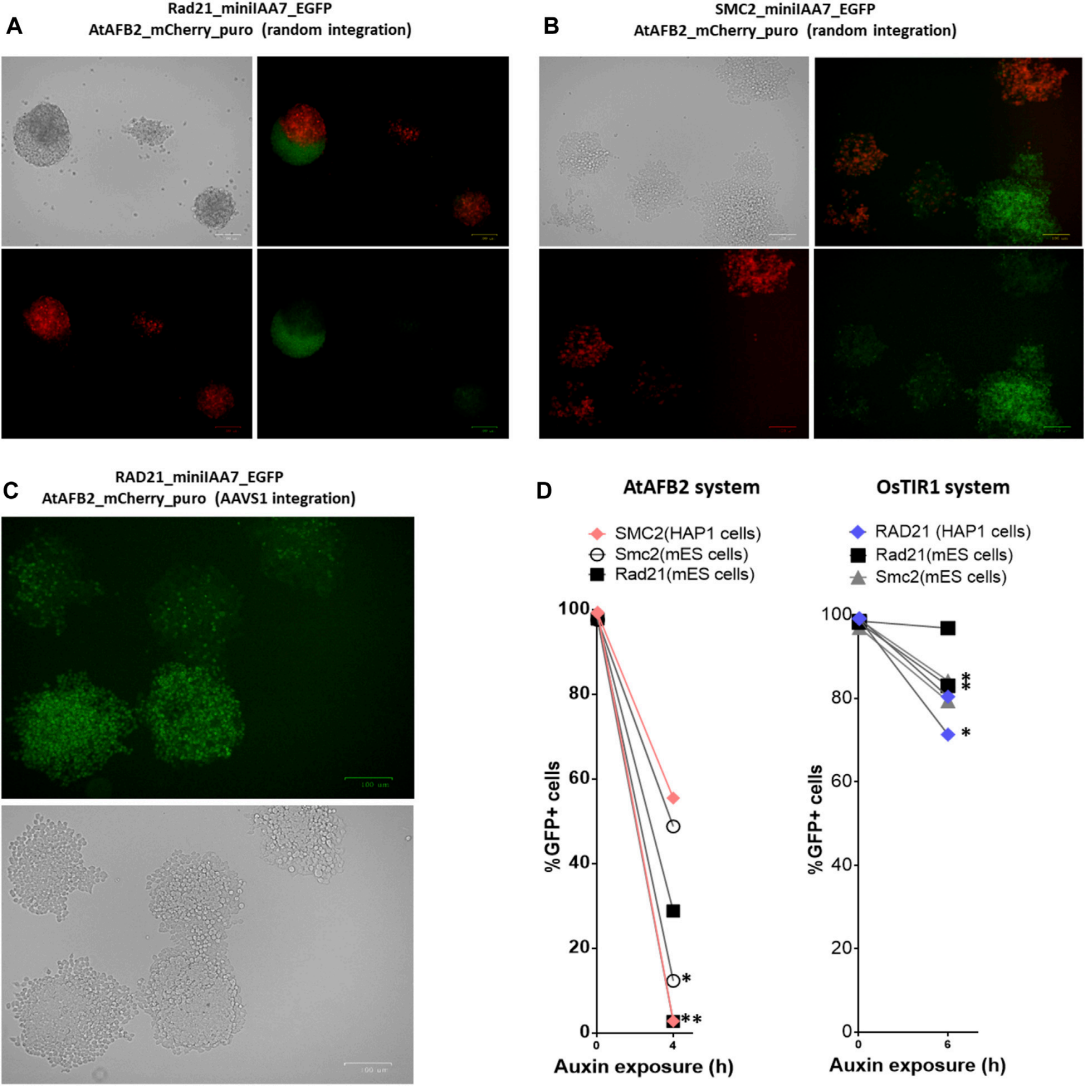
Mosaic expression of AFBs in mESCs and HAP1 cells. One-cell derived colonies of mES cells **(A)** and HAP1 cells **(B)** bearing the AtAFB2 expression cassette at random genomic loci after overnight auxin exposure. **(C)** One-cell derived colonies of HAP1 cells with AtAFB2 cassette integration into the AAVS1 locus after overnight auxin exposure. **(D)** The addition of puromycin to the culture medium (*) restores the protein degradation efficiency in mosaic clones. For each clone, the experiment was carried out once.

Although our initial experiment with the AtAFB2 AID system demonstrated satisfactory results (Figure 3), we observed that a subset of cell population remained refractory to auxin and its proportion increased with passages in culture (data not shown). Apparently, the decline of POI degradation activity stems from the silencing of AFB constructs. Rad21-miniIAA7-eGFP mES cells and SMC2-miniIAA7-eGFP HAP1 cells bearing randomly integrated AtAFB2 constructs were seeded with low density to generate single-cell founder colonies. After overnight treatment with auxin, we observed both eGFP-positive and -negative colonies (Figure 5B). Furthermore, we detected a significant number of eGFP/Cherry mosaic colonies (Figures 5A,B), even though all colonies were derived from single progenitors. Generally, cells with silenced AtAFB2-Cherry construct retained eGFP fluorescence (Figures 5A,B). For example, in Figure 4A, Cherry^pos^ part of the mESC colony successfully degraded Rad21-miniIAA7-eGFP and started to die, while remaining cells kept expressing Rad21-miniIAA7-eGFP in the absence of AtAFB2-Cherry.

It is well known that random integration of transgene leads to variable expression due to position effects and silencing. Integration of the *OsTIR1* and *AtAFB2* transgenes in the *AAVS1* safe-harbor locus in human cells is considered a sufficient measure to provide stable transgene expression (Natsume et al., 2016; Li et al., 2019; Sathyan et al., 2020). We inserted the AtAFB2 transgene into the *AAVS1* locus in one of the HAP1 lines with Rad21-miniIAA7-eGFP modification. The AtAFB2 construct has an in-frame *Cherry* selection marker, and thus, we were able to analyze AtAFB2 expression levels using FACS. Interestingly, all selected Cherry^pos^ clones with correct *AtAFB2* insertion were mostly mosaic in expression with 16% Cherry^pos^ cells at best (Supplementary Table S1). None of the six clones showed efficient POI depletion in further tests (data not shown). We repeated the experiment with one-cell founder colonies for one of these clones and observed the same outcome with mosaic colonies (Figure 5C).

Thus, in our experiments, targeted integration of the AFB into the *AAVS1* safe-harbor locus did not yield any significant advantages over random integration of AFBs. In addition, cells were heterogeneous in AFB expression and the ability to degrade AID-tagged protein after auxin exposure.

Our results show that some cells fail to facilitate degradation of target proteins due to the low expression of AtAFB2 protein. Therefore, it is important to keep cells under constant puromycin selection during multiple passages in culture or re-freezing. To demonstrate this, we cultured mES cells bearing *Rad21* and *Smc2* modifications (AtAFB2 and OsTIR1 systems) and HAP1 cells with *RAD21* modifications for the OsTIR1 system and *SMC2* modifications for the AtAFB2 system in the presence of 2 µg/ml puromycin for several passages and then analyzed target protein degradation dynamics after auxin treatment (Figure 5D). As expected, the efficiency of protein degradation was extremely improved in puromycin-treated cells but only for the AtAFB2 system (Figure 5D).

### Culture Medium pH Levels Affect AtAFB2-Induced Protein of Interest Degradation

**FIGURE 6:**
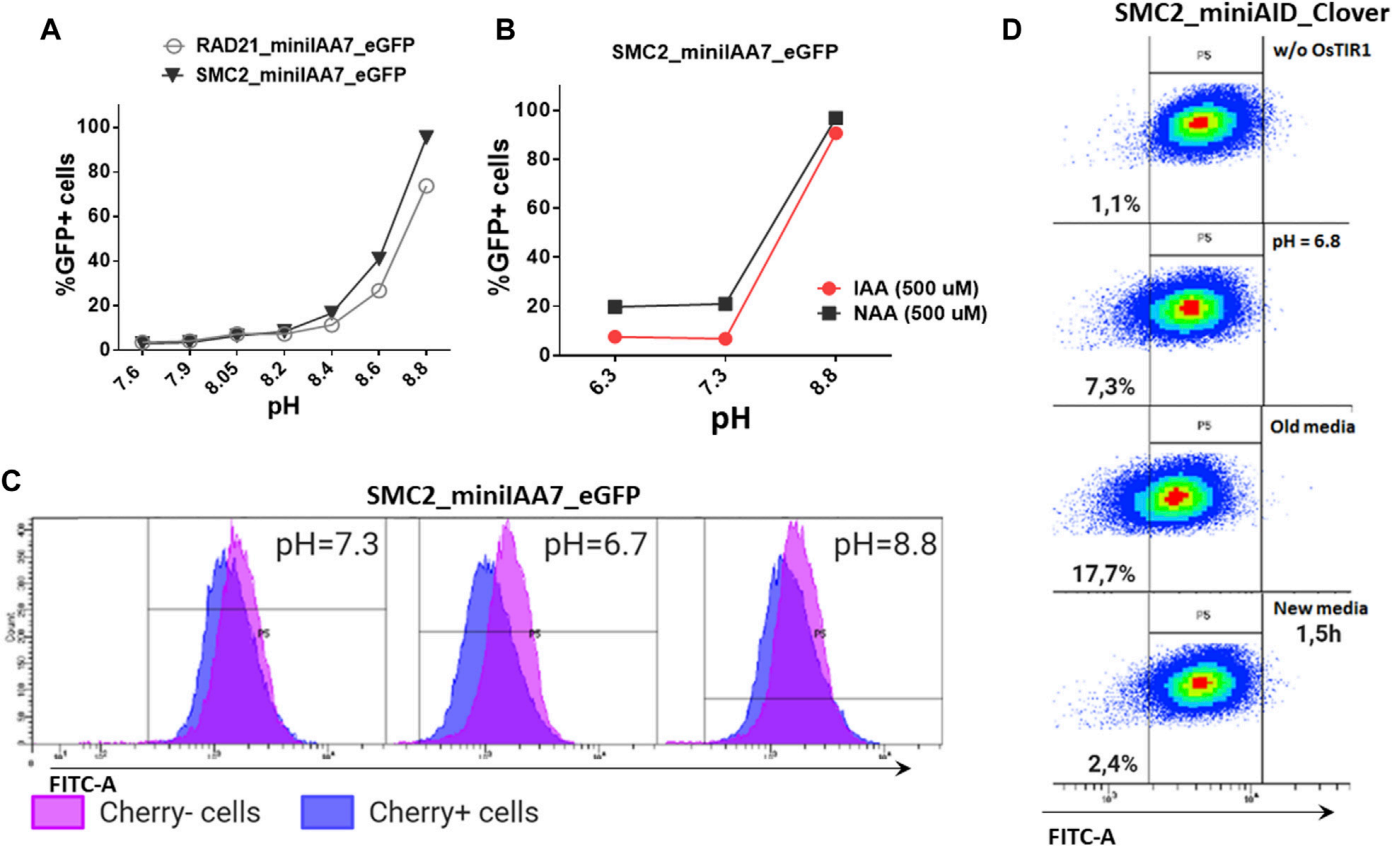
Effects of pH on AtAFB2 and OsTIR1 AID degradation efficiency. **(A)** High pH levels inhibit auxin-induced eGFP degradation in HAP1 clones with *RAD21* and *SMC2* modifications. Experiments were performed in duplicate. **(B)** IAA and NAA auxin analogs show similar sensitivity to pH changes (HAP1 clones). Experiments were performed in duplicate. **(C)** eGFP levels in the mosaic HAP1 clone SMC2-miniIAA7-eGFP. Blue color indicates eGFP levels in Cherry^pos^ subpopulation, and purple color indicates Cherry^neg^ subpopulation. The shift between two peaks illustrates minor basal degradation induced by AtAFB2 in Cherry^pos^ cells. **(D)** Shift of mES cell population (OsTIR1 system) toward left on the FITC-A axis in acidic media.

We also inspected whether pH level changes can affect POI degradation efficiencies. Acidification of culture medium pH (6.8 and below) is typically observed in prolonged incubations or in culture mediums with low buffering capacities (Michl et al., 2019). We were gradually changing pH values of the medium used for cultivating HAP1 RAD21/SMC2-miniIAA7-eGFP clones and later detected eGFP fluorescence on FACS, similar to previous experiments. Normal and mildly acidic pH (6.3–7.4) did not affect POI degradation efficiency, while higher pH values (8.6–8.8) effectively blocked POI degradation (Figures 6A,B). The auxin analog NAA (1-naphthaleneacetic acid) showed similar pH sensitivity (Figure 5B). Therefore, physiological pH changes in the cell culture medium do not affect auxin-dependent protein degradation efficiency.

Nevertheless, low pH might influence basal degradation in the AtAFB2 AID system. We inspected the mosaic HAP1 clone with miniIAA7-tagged SMC2, which consists of mixed Cherry^pos^/Cherry^neg^ subpopulations, caused by the silencing of the AtAFB2 expression in some cells (Figure 5B). Generally, Cherry^pos^ cells have a slightly weaker eGFP fluorescence profile [blue (Figure 6C)] with a shift between two peaks, probably reflecting minor basal degradation. At normal pH or high pH (no AtAFB2 activity), eGFP level shifts remain similar. However, changing the pH value to 6.7 led to a slight decrease in eGFP fluorescence in Cherry^pos^ cells, but not in Cherry^neg^ cells (Figure 6C, middle), which might hint at increased basal degradation at low pH. We observed the same slight shift for the OsTIR1 AID system while culturing cells in the acidic pH medium, including culture media in which cells were growing overnight (old media) (Figure 6D).

## Discussion

The auxin-inducible degron (AID) system consists of two molecular components. To begin with, a gene of interest has to be tagged with the AID domain and selection cassette with one of the two markers for double selection (e.g., *NeomycinR*/*HygromycinR*) (Natsume et al., 2016). Obtaining homozygous clones using double selection is convenient and highly efficient. In our experiments, for all six loci tagged (*RAD21*, *SMC2*, *Rad21*, *Smc2*, *Ncaph*, and *Ncaph2*), the overall efficiency ranged from 11 to 70% (Table 1). The only inconvenience that we faced was spontaneous diploidization of HAP1 cells (Figure 2). To avoid problems with obtaining homozygous integrations, it is important to select haploid clones by visual inspection of colonies or by FACS (Beigl et al., 2020). Additionally, one should check the copy number of the target loci in advance using real-time PCR or analogous methods because chromosome number may vary between HAP1 clones (Olbrich et al., 2017).

Similar to any other protein tag (Saiz-Baggetto et al., 2017; Booth et al., 2018), adding an AID-GFP tag to the N- or C-terminus could lead to destabilization of the protein or affect a critical functional domain (Morawska and Ulrich, 2013; Lu et al., 2021). Traditionally, placing the tag at the C-terminus is recognized as the safest approach, preserving upstream untranslated region (UTR) sequences. However, sometimes the C-terminal AID tag could also affect the expression due to the changes in the 3′ UTR (Yesbolatova et al., 2020). In addition, AID-tag composition (e.g., inclusion of the eGFP/mCherry protein) could influence degradation efficiency in some cases (Li et al., 2019). Due to these reasons, placement and composition of the AID-tag should be evaluated experimentally for each specific case, using western blotting or functional protein analysis. Extensive experiments with tagged components of the SMC complexes (cohesin, condensins, SMC5-6) (Gibcus et al., 2018; Venegas et al., 2020; Yesbolatova et al., 2020) show that C-terminal modification does not disrupt these proteins’ functions.

The key step to successful AID experiments is high and stable AFB (receptor F-box proteins, i.e., OsTIR1 and AtAFB2) expression. Our pilot experiments with the mAID/OsTIR1 system involved six proteins of interest (POIs) (Figure 1) and followed a popular protocol (Natsume et al., 2016). Using this approach, we obtained many clones with correct gene modifications (Table 1) but never succeeded in getting reliable degradation with the CMV-OsTIR1-Puro expression cassette (Figures 3A,B). One possible explanation for this may be the propensity of OsTIR1 to higher leaky degradation than AtAFB2 even without IAA (Li et al., 2019; Yesbolatova et al., 2019). Our experiments used the essential cell viability proteins Rad21 and Smc2, without which cells cannot survive for a long time. It can be assumed that the selection of cells took place, and those cells that could potentially effectively degrade the target protein died due to higher leaky degradation. Only clones having the lower expression level of AFB protein survived because they escaped from the lethality caused by the leaky degradation. A practical recommendation, in this case, may be the use of an inducible expression of the AFB protein (Natsume et al., 2016).

However, it should be emphasized that leaky degradation fails to explain the problems with the Ncaph2 protein. Cells with a knockout of this protein are viable (Hoencamp et al., 2021); nevertheless, our attempts to obtain cells with effective degradation of Ncaph2 on the OsTIR1 system were unsuccessful.

Most experiments on cultured cells used random OsTIR1 integration (Holland et al., 2012; Lambrus et al., 2015; Nora et al., 2017; Gibcus et al., 2018; Nakano et al., 2019). In theory, integration of the AFBs in the safe-harbor locus should provide sufficient expression levels. This approach is recommended in majority of protocols where many suitable loci (*H11*, *TIGRE*, *AAVS1*, and *Rosa26*) were extensively characterized (Baker et al., 2016; Nora et al., 2017; Pryzhkova et al., 2020). The downsides of the safe-harbor targeting approach are extra experimental manipulations, restricted expression levels, and susceptibility to silencing, just as in randomly integrated transgene constructs (Figure 5). For instance, *TIGRE* safe-harbor locus expression may vary after differentiation (Nora et al., 2017), which is critical for ES cell experiments. During our experiments, we tested both of these approaches and found no significant difference between targeted and random integration. Therefore, generating a set of clones with highly expressed randomly integrated AFB transgenes might be more convenient while providing clones with diverse expression levels suitable for any specific POI.

Surprisingly, we found that selected clones with efficient POI degradation became refractory to auxin after some time in culture due to AFB silencing (Figure 5). For example, we observed the mosaic mESC colony where roughly half of the cells lost Cherry expression (low AtAFB2) and could not degrade Rad21-miniIAA7-eGFP (Figures 5A,B). Mosaic silencing happens almost inevitably in most clones, which is unacceptable for depletion endeavors requiring 0–10% POI levels. Continuous supplementation of puromycin to the culture medium prevents AFB silencing in further experiments (Figure 5D). We feel that this fact is either omitted or not stressed enough in most published protocols (Natsume et al., 2016; Lambrus et al., 2018; Yesbolatova et al., 2019; Sathyan et al., 2020). Our observations also warn against removing the selection cassette after AFB integration in safe-harbor loci (Pryzhkova et al., 2020). In the future, exclusion of the AFB expression step could help to improve the AID experience (Yamanaka et al., 2020), but the method is still under development.

Comparing the two AID systems directly in our case could be difficult because AFB expression vectors differ between protocols (Natsume et al., 2016; Li et al., 2019). AtAFB2 has an in-frame fluorescent Cherry reporter useful for selection of high AFB-expressing clones during visual colony examination. However, it does not prevent silencing of the Cherry-expressing cells later (Figure 5). In the AtAFB2 construct, the *PuroR* gene is linked to the AFB via IRES, while in the CMV-OsTIR1-Puro vector, *PuroR* expression is driven from an independent promoter (Figure 1). The latter could hinder puromycin selection. Nevertheless, after AFB transfection and one-week puromycin selection, AtAFB2 clearly wins over OsTIR1 in terms of POI degradation efficiency (Figures 3A,B). Other factors, such as the absence of basal depletion and degradation dynamics, were also in favor of the AtAFB2 system (Figures 3C–E,6C). A recent study reports that basal degradation in the OsTIR1 AID could be countered by overexpressing ARF protein (Sathyan et al., 2020). Unfortunately, this approach is not applicable for the popular miniAID (mAID) system because the mAID lacks ARF-interacting domains (Sathyan et al., 2020).

It is known that the binding affinity of auxin with its receptors would be pH-dependent (Badescu and Napier, 2006). To our knowledge, how pH levels affect auxin-dependent degradation in cultured mammalian cells was never reported before. We showed in HAP1 cell lines that increasing pH from 7.4 to 8.8 completely blocks AtAFB2-dependent degradation, while the decrease of pH does not affect AtAFB2 (Figure 6). Such pH-based switch could be a simple lever to temporarily block POI degradation during experiments. AID pH sensitivity might be worth testing in the context of the dual degradation approach like in an alternative coronatine-dependent JAZ degron/COI system combined with OsTIR1 (Brosh et al., 2016; Sybirna et al., 2020).

## Materials and Methods

### Cell Culture

All cell lines were grown at 37°C with 5% CO_2_ and passaged every 2–3 days. HAP1 cells (Horizon Discovery) were cultured in IMDM (Thermo Fisher) containing 10% FBS (Gibco), 1 mM L-glutamine (Sigma), 0.5 mM NEAA (Gibco), and penicillin/streptomycin (100 U ml^−1^ each). Mouse ES cells (DGES1 line) established in our laboratory (Kontsevaya et al., 2017) were cultured on a gelatin surface under 2i conditions (1 μM PD, 3 μM CHIR) in DMEM (Thermo Fisher), supplemented with 7.5% ES FBS (Gibco), 7.5% KSR (Gibco), 1 mM L-glutamine (Sigma), NEAA (Gibco), 0.1 mM *β*‐mercaptoethanol, LIF (1000 U/ml, Polygen), and penicillin/streptomycin (100 U ml^−1^ each). 3-Indoleacetic acid (IAA, auxin) (Merck, I2886) and 1-naphthaleneacetic acid (NAA) (Merck, N0640) were dissolved in NaOH (1M) solution to a concentration of 500 mM, aliquoted, stored at -20°C, and used immediately after thawing. For all experiments, the cells were incubated with 500 μM indole-3-acetic acid (IAA) (Sigma) for the indicated times.

For testing the pH-responsive auxin-inducible degradation of POI, cell culture media with a pH range of 6.3–8.8 were prepared and used immediately. The cells were harvested no longer than 30 min post auxin exposure because alkaline pH rapidly returns to neutral values in CO_2_-rich atmosphere.

### Construction of the Auxin-Inducible Degron Donor Vectors and Site-Specific Degron Integration

Donor vectors with homology arms, degron tag (mAID or miniIAA7), Clover/eGFP, and selection cassette (either NeoR or HygroR) were assembled in one reaction using the NEBuilder HiFi kit (NEB) in the pMK289 backbone (Natsume et al., 2016). Homology arms for *RAD21* (NCBI Entrez Gene ID: 5885), *SMC2* (NCBI Entrez Gene ID: 10592), *Rad21* (NCBI Entrez Gene ID: 19357), *Smc2* (NCBI Entrez Gene ID: 14211), *Ncaph* (NCBI Entrez Gene ID: 215387), and *Ncaph2* (NCBI Entrez Gene ID: 52683) genes were PCR amplified from human and mouse genomic DNA with Q5 polymerase (NEB). The lengths of homology arms are featured in Supplementary Table S2. Other functional elements were amplified from plasmids pMK289 (mAID-mClover-NeoR) (Addgene #72827), pMK290 (mAID-mClover-HygroR) (Addgene #72828), and pSH-EFIRES-B-Seipin-miniIAA7-mEGFP (Addgene #129719) (Natsume et al., 2016; Li et al., 2019). For auxin receptor F-box protein (AFB) overexpression, OsTIR1 and AtAFB2 plasmids were used (AtAFB2) (pMK232 CMV-OsTIR1-PURO, Addgene #72834; pSH-EFIRES-P-AtAFB2-mCherry-weak NLS, Addgene #129717) (Natsume et al., 2016; Li et al., 2019). For gene tagging and AAVS1 locus modification, the cells were transfected with donor vectors, Cas9 (Addgene #41815), and corresponding sgRNA-expressing plasmids (Addgene #41824, backbone). In order to precisely replace the AtAFB2 gene in the pSH-EFIRES-P-AtAFB2-mCherry-weak NLS (Addgene #129717) vector with the *OsTIR1* gene, we assembled two PCR-amplified fragments (the *OsTIR1* gene and the *Cherry* gene) with the BglII/NotI linearized backbone using NEBuilder HiFi DNA Assembly Master Mix. Sequences of sgRNAs are provided in Supplementary Table S2.

### Generation of Transgenic Cell Lines With Auxin-Dependent Degradation of Protein of Interest

HAP1 cells were electroporated at conditions of 1200 V, 30 ms, 1 pulse using the Neon Transfection system (Invitrogen), according to the manufacturer’s instructions. The day after, electroporation cells were seeded in 100-mm dishes with low density and expanded for 24 h, followed by selection with 500 μg/ml G418 (for *RAD21* targeting) or 800 μg/ml hygromycin B (for *SMC2* targeting). Mouse ES cells were transfected by Lipofectamine 3000 (Invitrogen) in OPTI-MEM (Thermo Fisher), following the supplier’s recommendations. After 24 h, transfection cells were seeded in 60-mm dishes in the culture medium containing 300 μg/ml G418 and 200 μg/ml hygromycin B for selection of homozygous tagged clones (Natsume et al., 2016). After 10–14 days of selection, resistant colonies of HAP1 cells and mouse ESCs expressing green fluorescent protein (eGFP) were manually picked for subcloning and further analysis. Most of the surviving clones were confirmed to harbor the correct insertion of degron tags by genomic PCR, Sanger sequencing, and western blotting. In addition, the resulting HAP1 clones were stained with propidium iodide (PI), and their DNA ploidy was determined by FACS (Beigl et al., 2020). Several clones for each target gene were chosen for integration of AtAFB2- or OsTIR1-expressing vectors and transformed using identical settings described for the previous step. Selection of the AFB-positive clones was performed using 1 μg/ml puromycin for 1 week. Finally, several mESCs and HAP1 clones with homozygous degron tags and AFB insertions were tested for efficiency of auxin-induced protein depletion, using microscopy, FACS, and western blotting.

### Polymerase Chain Reaction Genotyping

The cells were lysed in PBND lysis buffer (10 mM Tris-HCl, 50 mM KCl, 2.5 mM MgCl_2_, 0.1 mg/ml gelatin, 0.45% NP-40, and 0.45% Tween 20, pH 8.3) containing proteinase K (200 μg/ml) for 1 h at 55°C followed by proteinase K inactivation for 10 min at 95°C. The target regions were amplified by PCR with either Taq-HP DNA Polymerase (Biospecifika Novosibirsk, Russia) or LongAmp Taq DNA Polymerase (NEB) (primers sequences could be found in Supplementary Table S3). The parameters were as follows: 95°C for 30 s, then 35 cycles of 94°C for 10 s, 60°C for 30 s, 72°C for 1 min (1 min/kb), and a final step at 72°C for 5 min for Taq-HP DNA Polymerase and 94°C for 30 s, then 30 cycles of 94°C for 10 s, 60°C for 30 s, 65°C for 3–6 min (1 min/kb), and a final step at 65°C for 5 min for LongAmp Taq DNA Polymerase. The amplified products were analyzed by agarose gel electrophoresis.

### Western Blotting

The cells were washed twice with PBS and scrapped from the surface in the presence of RIPA buffer (50 mM Tris-HCl pH 8, 150 mM NaCl, 1% Triton X-100, 0.5% sodium deoxycholate, and 0.1% SDS) containing the protease inhibitor cocktail [1x Complete ULTRA (Roche), 1x PhosSTOP (Roche), 5 mM NaF (Sigma)]. After that, the cells were sonicated by three 10 s pulses at 33–35% power settings. Lysates were centrifuged at 18,000 g for 20 min at 2°C, frozen, and stored at -80°C. The protein concentrations in cell lysates were quantified using Pierce BCA Protein Assay Kit (Thermo Fisher). Equal amounts (25 μg) of total protein were separated on 10% SDS-PAGE and then transferred onto the Immun-Blot PVDF membrane (Bio-Rad). After blocking in 5% milk/TBST for 2 h, the membrane was incubated with primary antibodies against RAD21 and SMC2 (#12673/#8720, Cell Signaling Technology) at 4°C overnight. On the following day, membranes were incubated with horseradish peroxidase–conjugated secondary antibodies (#7074, Cell Signaling Technology) for 2 h at 25°C. Detection was performed with SuperSignal West Pico PLUS Chemiluminescent Substrate (#34580, Thermo Fisher) and ChemiDoc XRS Imaging System (Bio-Rad).

GraphPad Prism 6.0 was used to design graphs and analyze quantitative data. The results are presented as mean values ± standard deviations.

## Conclusion

In conclusion, here we outlined the AID approach that showed success in our hands. Our experience was based on evaluation of the OsTIR1 and AtAFB2 systems in mES and human HAP1 cells, which might help readers avoid common pitfalls. We generated bi-allelic tag modifications first using CRISPR/Cas9 and miniIAA7 donor vectors with double selection markers, then selected a few clones validated with long-distance PCR and Sanger sequencing, and randomly inserted the AtAFB2 cassette with in-frame PuroR and Cherry selection markers. Usually, 10–20% of puromycin-resistant AtAFB2 clones display high POI degradation efficiency. It is advisable to always use puromycin selection during experiments to avoid AtAFB2 silencing.

## Data Availability

The original contributions presented in the study are included in the article/Supplementary Material, and further inquiries can be directed to the corresponding authors.
