# Supplementary material for: Evaluation of the OsTIR1 and AtAFB2 AID Systems for Genome Architectural Protein Degradation in Mammalian Cells
Source: Front Mol Biosci. 2021 Nov 4;8:757394. doi: 10.3389/fmolb.2021.757394 (PMC8599953; doi:10.3389/fmolb.2021.757394)
Supplement: Supplementary file 1 [file DataSheet1.PDF]

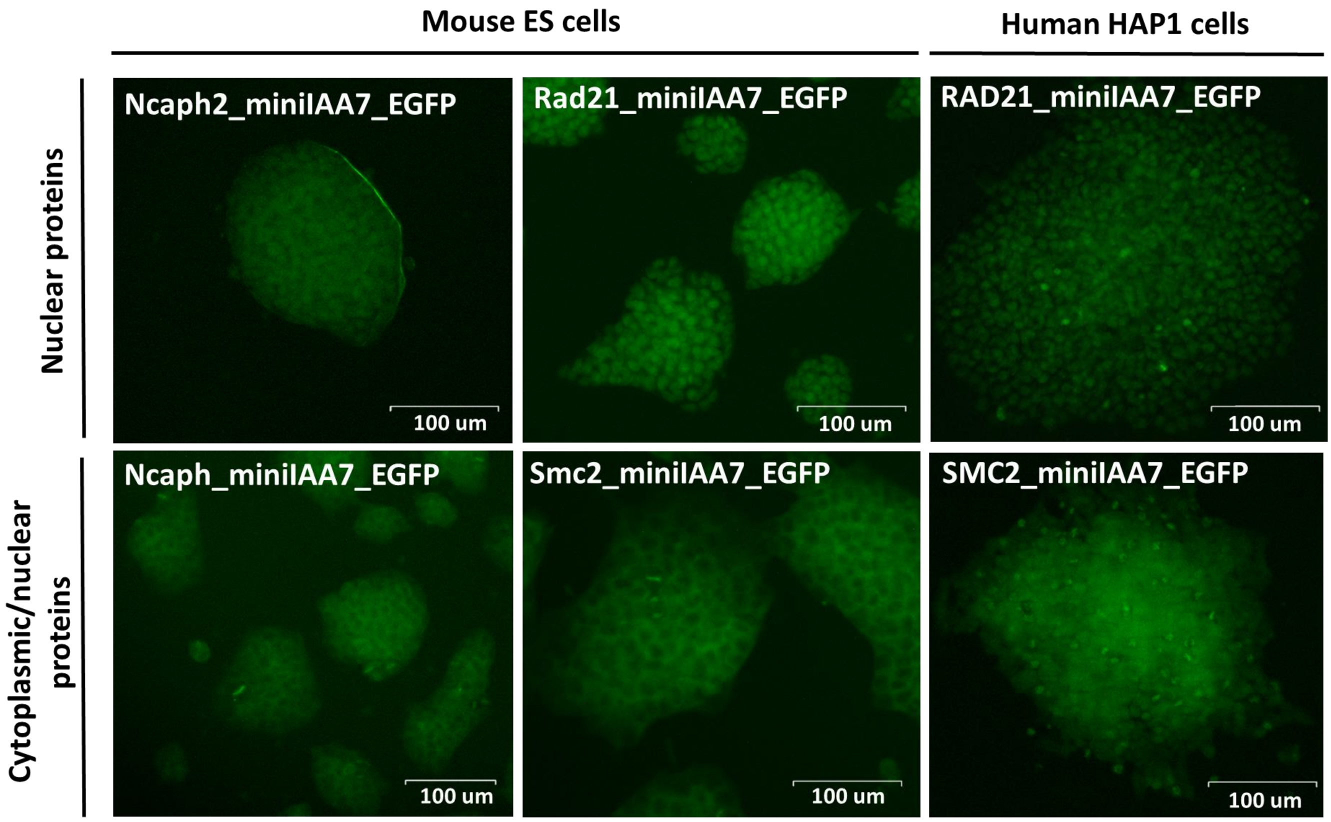

Supplementary Figure S1. Distribution of the POI-miniIAA7-eGFP protein in homozygous tagged mESCs lines and HAP1 lines. Top pictures: Nuclear localization (Rad21 and Ncaph2 clones). Bottom pictures: Predominantly cytoplasmic localization (Smc2 and Ncaph clones). Thick bands visible in some of the cells label mitotic spindles, which are covered with condensins during division.

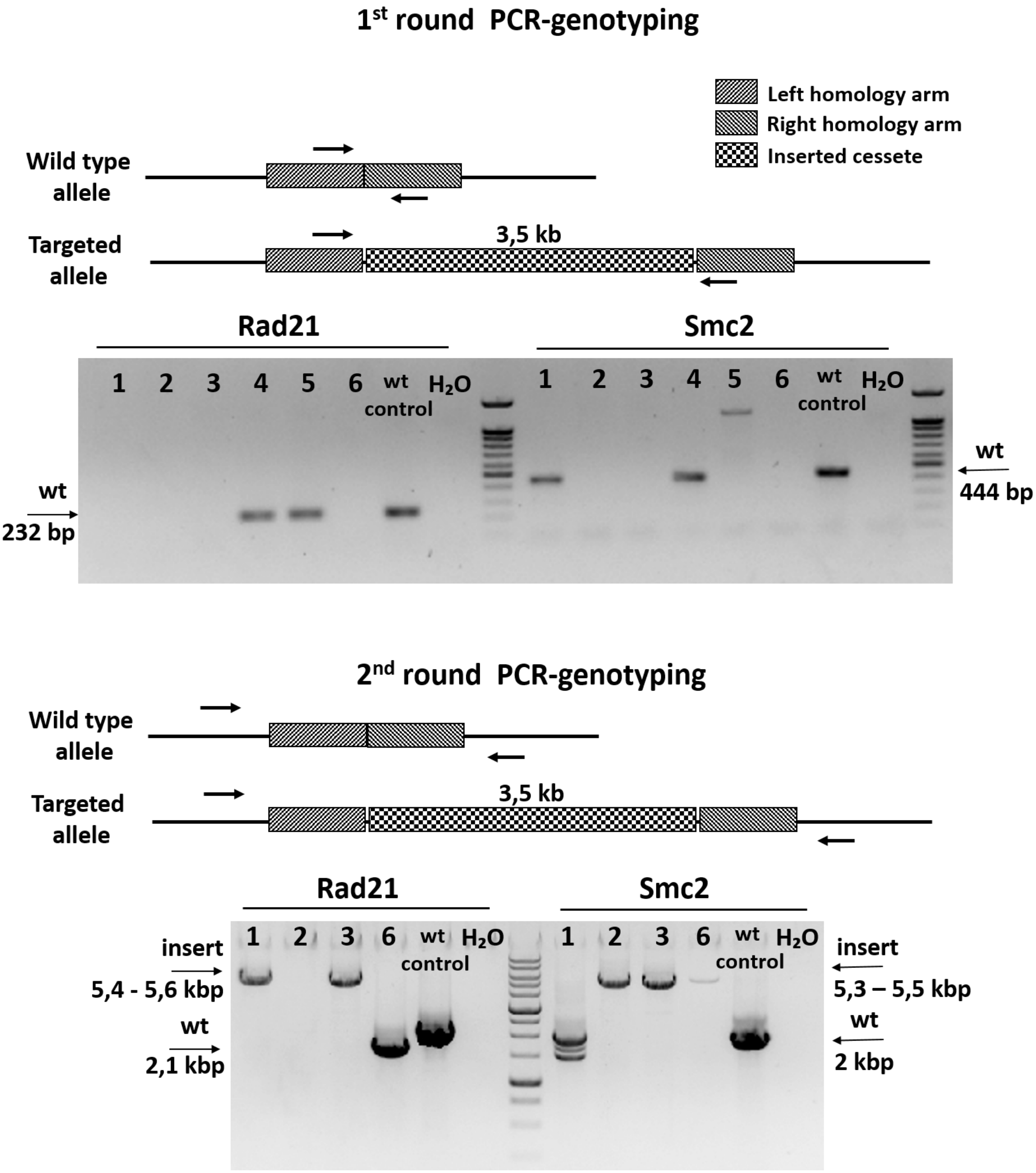

Supplementary Figure S2. PCR genotyping strategy for selecting correctly AID tagged genes (Rad21 and Smc2 mESC clones; miniIAA7 tag). 1st round: primers complimentary to the modified loci amplify wild-type (wt) alleles (300-500 bp). The lack of amplicons indicates either targeted cassette insertion or the large deletions involving the primer-binding sites. 2nd round: primers beyond the donor plasmid homology arms were used in long-distance PCR (5-6 kbp).

Indicated amplicon sizes confirm the correct integration of constructs. Amplicons for wt and targeted alleles (AID cassette insertion) are marked with arrows. Apparently, some wt alleles carry deletions at the modified loci (Rad21 N2,6; SMC2 N1).

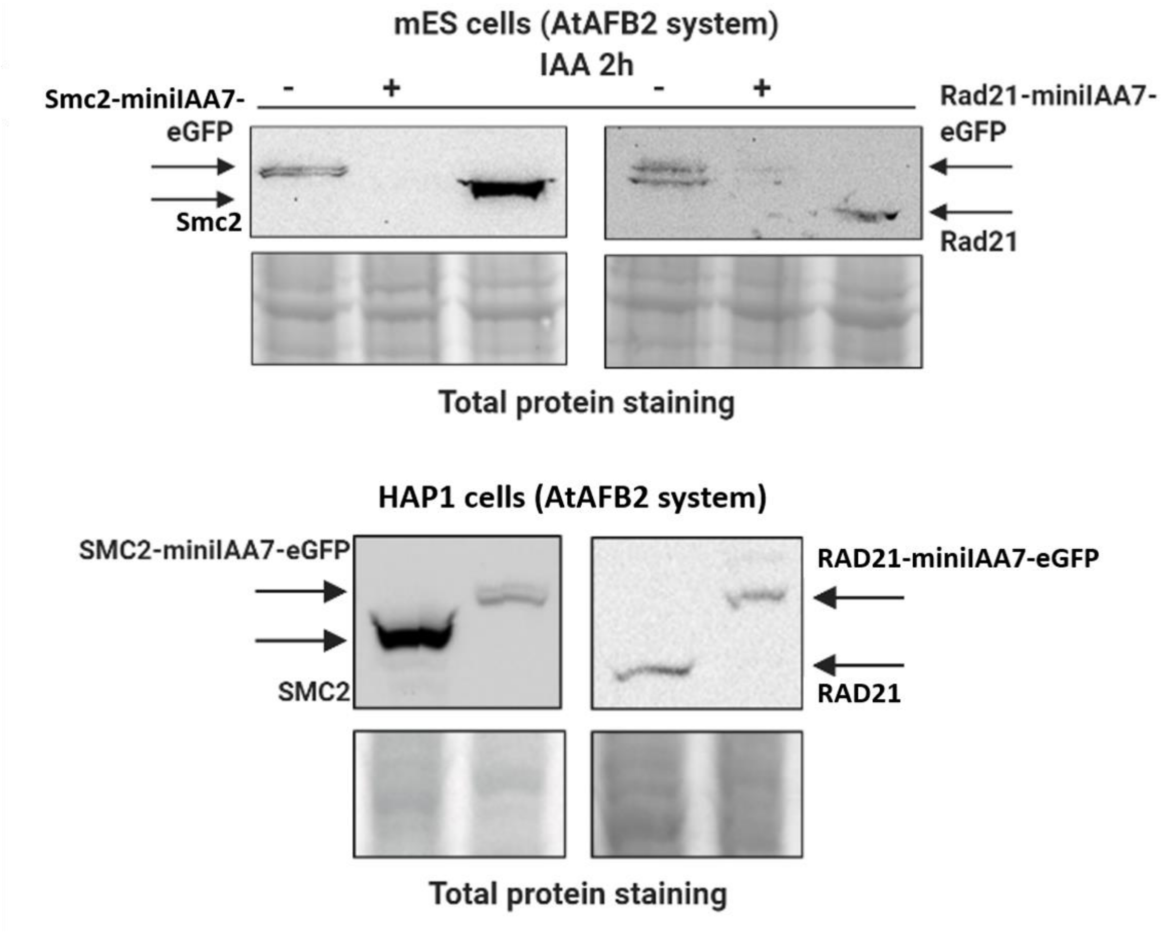

Supplementary Figure S3. Western blot analysis for Smc2 and Rad21 proteins before and after insertion of degron tags in mESCs and HAP1 cell clones. (+) - degradation of POI-miniIAA7-eGFP after addition of auxin (IAA). (-) - no auxin.

Supplementary Table S1. Integration of the AtAFB2 in the AAVS1 locus results in mosaic clones (%, Cherry) with low degradation efficiency.

Clone number	Ploidy	%, Cherry+ cells	AAVS1 integration
20	hap	16,2	+
34	hap	12,5	+
54	hap	12,4	+
58	hap	6,3	+
64	hap	6	+
72	hap	1,9	+

Supplementary Table S2. Sequences of sgRNAs and donor vector arms lengths used in AID targeting experiments.

Gene target	sgRNA sequence (5′-3′)	5H homology arm	3H homology arm
mES-*Rad21*	ccacggttccatattatctg	681 bp<br>mm10 Chr15:51828135-51827455	1270 bp<br>mm10 Chr15:51827452-51826183
mES-*SMC2*	tgaactggcatgcactcagt	707 bp<br>mm10 Chr4: 52485946-52486652	1061 bp<br>mm10 Chr4: 52486656-52487716
mES-*Ncaph*	ccgctgcagacgtctcaaag	1031 bp<br>mm10 Chr2: 126947174-126946144	962 bp<br>mm10 Chr2: 126946135-126945174
mES-*Ncaph2*	ctgatgttctggtgatgcaa	661 bp<br>mm10 Chr15:89254991-89255651	1008 bp<br>mm10<br>Chr15:89255655-89256662
Hap1-*RAD21*	ccaaggttccatattatata	780 bp<br>hg38 Chr8: 116848282-116847503	1232 bp<br>hg38 Chr8: 116847499-116846268
Hap1-*SMC2*	accacccaaaggagcacatg	686 bp<br>hg38 Chr9: 104138627-104139312	1036 bp<br>hg38 Chr9: 104139316-104140351
AAVS1	ggggccactagggacaggat	804	837

Supplementary Table S3. Oligonucleotides used for genotyping of modified clones.

Gene/Site	Direction	Sequence (5′-3′) 1st round PCR-genotyping	2nd round PCR-genotyping
mES-*Rad21*	F	tgcttgagctgtgtcgaaacac	acccaataagcgacaaaaactgg
	R	agggttttggtgccctgc	atgaagtgtcaagccagggg
mES-*Smc2*	F	gtggtatccctcaaagaaggtatg	gtgtagttggccaagtttcctc
	R	gggcatttgcatacacaatttacc	aatgttccagggtacaaatgactt
mES-*Ncaph2*	F	gcaactggtaagtggcctg	gcaggaggggagaatgtagc
	R	tccacagtgtcttgccctg	tgaatgtggggcagatgctt
mES-*Ncaph*	F	gccacgagatgattctccttg	gacaagagtgtgcaaagcgg
	R	aacagcatggagcttcctagc	catcatcccaggtgctgacc
Hap1-*RAD21*	F	cagcgtgctcttgctaaaact	caactcctttgtatgcctgcac
	R	aagattgccagtgttactgatggaa	aacggggaaaactccttggtt
Hap1-*SMC2*	F	caagcagtcaaccaccagga	catgtgacacttgatgggga
	R	tcacaaccacaataattggaccat	gtacggccatatatcagggga
AAVS-1	F	tccaggggtccgagagctcagctag	
	R1	cgggattctcctccacgtcaccgca	
	R2	ccagacagccgcgtcagagcagctc	
